# Redefining fan manufacturing: Unveiling industry 5.0's human-centric evolution and digital twin revolution

**DOI:** 10.1016/j.heliyon.2024.e33551

**Published:** 2024-06-26

**Authors:** Taoer Yang, Luqman Razzaq, H. Fayaz, Atika Qazi

**Affiliations:** a School of Law, Xiamen University, Fujian, China; b Department of Mechanical Engineering Technology, University of Gujrat, Gujrat, 50700, Pakistan; c Modeling Evolutionary Algorithms Simulation and Artificial Intelligence, Faculty of Electrical & Electronics Engineering, Ton Duc Thang University, Ho Chi Minh City, Viet Nam; d Centre for Lifelong Learning, University Brunei Darussalam, Brunei Darussalam

**Keywords:** Industry 5.0, Digital twins, Fan manufacturing, Sustainable, Human centric, Resilience

## Abstract

Industry 5.0 has the capacity to surpass the technology -oriented efficiency of Industry 4.0 and advance sustainable development objectives such as prioritizing human needs, ensuring socio-environmental sustainability, and enhancing resilience. Digital twins and simulation technologies improve manufacturing, evaluate products and operations, and predict any potential adverse consequences. With digital twin technology, everything that exists in the physical world will eventually be duplicated in the digital realm. Within the context of Industry 5.0, this study aims to investigate the impact of digital twin technology on the fan manufacturing sector. The proposal for implementing the enabling industry 5.0 application was presented to the chief engineers of eight distinct fan manufacturers. Out of these, five responded positively and their feedback was subsequently followed up on. Three different avenues such as production, supply chain, and testing transparency were proposed for industry 5.0 implementation. The exploration of testing transparency is being undertaken based on a consensual decision. The Web of Things standard that enables digital twin generation in industry 4.0 is implemented to enable the testing transparency. This data was linked with the internal digital twin of fan motor created using ANSYS. This digital twin can predict the lifespan of the motor by analyzing the temperature of the motor housing surface. Toward sustainability and resilience with Industry 4.0 and Industry 5.0 may provide insights into this alignment.

**Table:** Nomenclature

DT	Digital twin
IoT	Internet of things
FEA	Finite Element Analysis
FM	Fan Manufacturing
QoS	Quality of Service
RFID	Radio Frequency Identification
ML	Machine Learning
CPS	Cyber Physical System
AI	Artificial Intelligence
DFS	Distributed File Systems

## 1 Introduction

The introduction of "Industry 4.0″ has resulted in significant changes to the technologies used in the design and manufacture of complex technical items [1]. The phrase "Industry 4.0″ was initially offered to the public in 2011 by a consortium comprising members from Germany's business, political, and scientific sectors [2]. The term "cyber-physical systems" (CPS) integration was established as a method to enhance industrial competitiveness by incorporating CPS into manufacturing processes [3]. The existing body of literature recognizes that Industry 4.0 is primarily focused on enhancing productivity through the utilization of technology [4]. While Industry 4.0 unintentionally enhances certain micro-environmental sustainability indicators like production efficiency and pollution reduction, it is unable to transcend the profit-centric nature of current production and consumer economic models. The notion of Industry 5.0 has lately arisen as a visionary perspective on a future industrial sector that prioritizes the preservation of the environment and the well-being of society [5]. The proponents of Industry 5.0 assert that Industry 4.0 does not provide an adequate foundation for attaining sustainable growth [6].

The adoption of Industry 4.0 by organizations led to the birth of the Fifth Industrial Revolution, often known as Industry 5.0 [7]. Industry 5.0 acknowledges the capacity of the industrial sector to accomplish societal objectives that go beyond employment, and expansion, to transform into a resilient facilitator of economic well-being, through the act of production adheres to the limits of our world and prioritizes the welfare of the industrial laborer at the core of the manufacturing process [8]. Industry 5.0 is introduced under the premise that Industry 4.0 prioritizes digitalization and AI-driven technologies to enhance production efficiency and flexibility, rather than emphasizing the fundamental ideals of social fairness and sustainability [[9], [10], [11]]. Therefore, Industry 5.0 offers a fresh perspective and new way of thinking about the industry's role in supporting people within the planet's long-term service needs through research and innovation [12].

Industry 5.0 is an approach to rethinking the future of energy, production, transportation, and supply networks that expands upon and enhances the substantial foundation laid by Industry 4.0's vision [13]. To humanize the idea of digital transformation, Industry 5.0 employs collaborative robotics and artificial intelligence [14]. Industry 5.0, supported by the European Commission and other governmental bodies, prioritizes a triple-bottom-line approach that considers the economic, environmental, and societal impact [10]. It aims to bring a perspective of ESG (Environment, Social, and Governance) and achieve a balance between technology-driven and economically focused decision [15]. The concept of Industry 5.0 has sparked considerable debate among scholars and professionals in the industrial sector. Researchers have provided multiple explanations for the widespread occurrence of Industry 5.0. In a study conducted by Özdemir and Hekim et al. [16], Industry 5.0 was described as a progressive advancement of Industry 4.0. This upgrade aims to provide balanced innovation to overcome the constraints of the industry 4.0 innovation ecosystem. In 2021, the European Commission unveiled its Industry 5.0 agenda, which aims to foster a resilient, sustainable, and human-centric European industry [17]. As per this agenda, Industry 5.0 is an additional effort that expands on the industry 4.0 framework to give priority to rising socio-environmental requirements.

Industry 5.0 is a fascinating socio-technological phenomenon. Industry 5.0 is a remarkable technological phenomenon, as it revolves around the progress of technology and the digitization of industrial value networks [18]. The industry of 5.0 is not only a social phenomenon, but it also relies on the culture of social dialogue among stakeholders [19]. This dialogue helps to effectively manage and guide technological innovation to promote important socio-cultural values such as human dignity, equality, privacy, and autonomy. Effective factory safety management is essential for supporting the implementation of human-centered manufacturing in the context of Industry 5.0 [20]. Conventional factory safety management relies on human expertise, including video surveillance, routine inspections, on-site safety signage, and so on [21].

Dr. Michel Grieves introduced the idea of digital twins in 2003 [22]. Initially, it was defined as "a collection of virtual information structures that provide a comprehensive description of potential or existing manufacturing products, ranging from the micro atomic level to the macro geometric level." Subsequently, certain academics characterized it as "the electronic portrayal of tangible entities or systems". In 2012, Glaessegen et al. provided a well-accepted definition of digital twins as a sophisticated simulation model of a product that incorporates several physical, scale, and probability factors [23]. This model is capable of accurately representing the real-time condition of actual products. The "2020 Digital Twins White Paper" was released by the New Generation Information Technology Industry Standardization Forum, sponsored by the China Electronics Technology Standardization Institute under the Ministry of Industry and Information Technology [22]. The paper was led by the Ministry of Industry and Information Technology. The utilization of modeling, simulation, data fusion, and other technologies is shown to enhance the development of digital twins [24]. The advent of the digital twins has led to a growing recognition of the significance of data and statistics, as well as the ability to accurately predict the behavior of physical objects. Consequently, individuals have the ability to circumvent superfluous hazards and exorbitant expenditures in the physical realm. Essentially, digital twins include using digital information to substitute real entities, allowing for an accurate representation of their value [25]. Digital twins offer enhanced solutions for the management, monitoring, and repair of many components of production machinery [26].

The present study is an effort to utilize digital twin technology in the fan manufacturing industry. The framework of industry 5.0 application was presented to the chief engineers of eight distinct fan manufacturers, resulting in five favorable responses that were then followed up on. Three distinct approaches, namely production, supply chain, and testing transparency, were suggested for the adoption of industry 5.0. The three distinct approaches encompassed production, supply chain, and testing openness. The exploration of testing transparency is being undertaken based on a consensual decision.

## 2 Industry 5.0 approach

Assessing any company's level of digital maturity is the initial stage in leveraging digital technology to drive value and achieve profitable business transformation. Therefore, it is crucial to adopt an Industry 5.0 maturity model to initiate organizational digital transformation [27]. Without comprehending organization present condition and devising a strategy, is just like to run the danger of making expensive however unproductive choices or investing in endeavors that current technology cannot accommodate. Digital maturity refers to the capacity to respond to promptly and effectively or capitalize on market opportunities, leveraging existing technological infrastructure, workforce capabilities, and digital tools [28]. A company's capacity to undertake digital transformation encompasses not just the adoption of digital technology, but also the integration of people, culture, and procedures across the entire organization [29]. The objective is to achieve business results while minimizing the potential for human error. The digital maturity of an organization is influenced not only by its focus on technology, but also by its speed and adaptability, which are primarily determined by the availability of skilled personnel and automated workflows [30]. It is a collaborative endeavor, in its most genuine form.

**Fig. 1:**
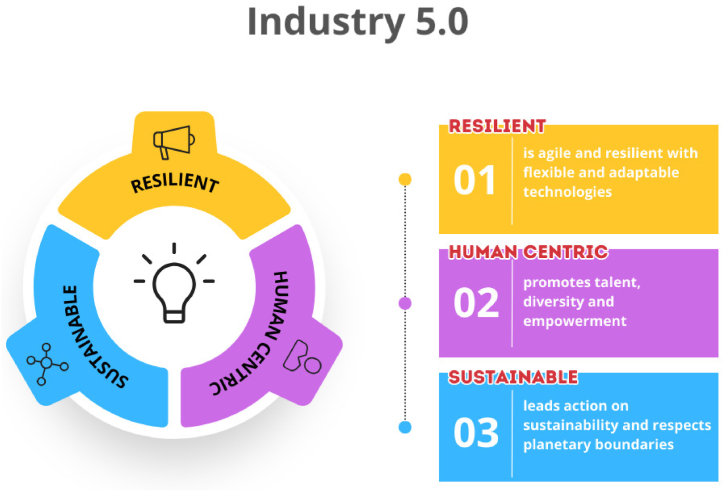
Fundamental principles of Industry 5.0.

Industry 5.0 is a conceptual framework that envisions the future of energy, manufacturing, mobility, and supply networks. It builds upon and complements the significant progress made by Industry 4.0. Industry 5.0 employs interactive robots and AI to incorporate a human element into the notion of digital transformation. Industry 5.0 revolves around three interrelated fundamental principles: human centric, promoting sustainability, and fostering resilience as shown in Fig. 1 [31].

The human-centric approach prioritizes the fundamental needs and interests of humans in the manufacturing process, moving away from a technology-focused approach and adopting a more human-centered and society-centered perspective [5]. Consequently, workers in the industry will assume new positions as the perception of their value changes from being seen as a "cost" to a "investment". The primary objective is to establish a secure and all-encompassing workplace that gives utmost importance to the physical health, mental health, and overall well-being of individuals, while also ensuring the protection of workers' fundamental rights, such as autonomy, human dignity, and privacy. Industrial workers must continuously enhance their skills and acquire new ones in order to improve their career prospects and achieve a better balance between work and personal life.

Resilience pertains to the necessity of enhancing the durability and strength of industrial production, making it more resistant to disturbances and capable of supplying and sustaining vital infrastructure during times of crisis. The future industry must possess the ability to adapt quickly to geopolitical changes and natural disasters.

### 2.1 Role of technologies in industry 5.0

**Fig. 2:**
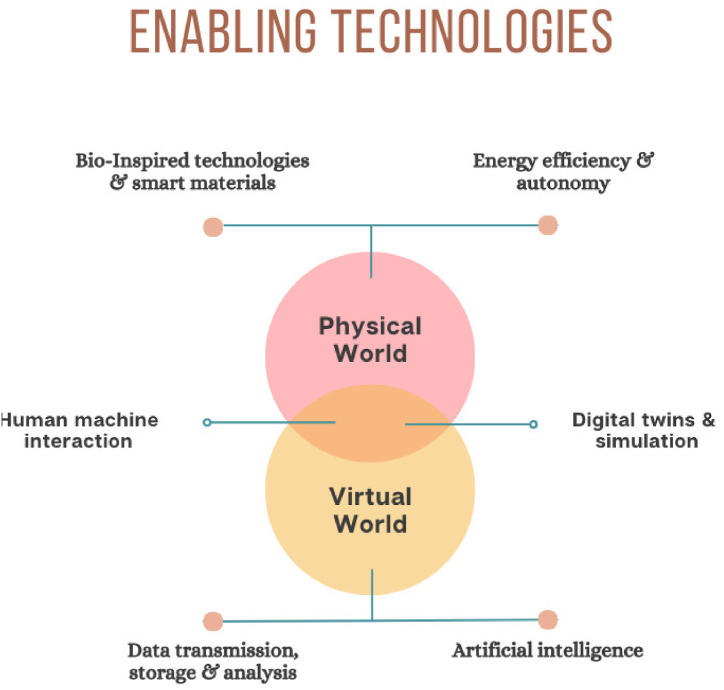
Enabling technologies of Industry 5.0.

The foundational technologies of Industry 5.0 encompass intricate systems that integrate advanced technologies, including innovative materials with embedded sensors inspired by biological processes. Hence, the full potential of each category can only be realized when integrated with others, inside systems and technological frameworks. The objective is to establish a connection between humans and technologies, providing assistance to humans and integrating human creativity with the capabilities of machines. The subsequent technologies facilitate humans in both physical and cognitive endeavors. Fig. 2 depicts the details of human support by different technologies.

### 2.2 Digital twins

Digital twins strive to create a comprehensive and autonomous reflection of the physical world in the digital realm using digital methods [25]. The mirror model enables a continuous and interactive connection with physical entities, allowing for a comprehensive understanding, analysis, and optimization of their entire life cycle. This is achieved through the use of simulation, verification, prediction, and control techniques, utilizing historical and real-time data, as well as algorithm models [32]. To put it more simply, digital twins are virtual copies of physical systems or devices. The primary distinguishing characteristic of this replica is its ability to accurately simulate the behavior of solid objects. Subsequently, digital twins are a continuously evolving procedure. The implementation of digital twins relies on the utilization of digital visual modeling and industrial simulation technology. Digital models facilitate the operation of physical entities, and conversely, physical entities influence digital models, so achieving a genuine merger of virtual reality through bidirectional mapping.

**Fig. 3:**
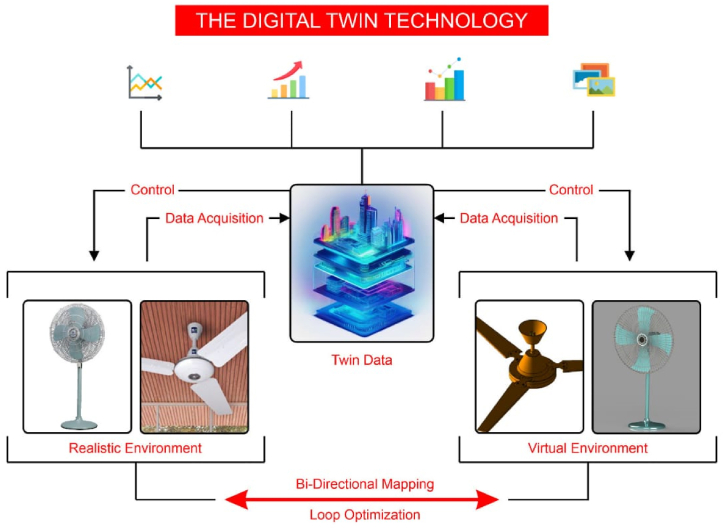
Utilization of digital twin technology in fan manufacturing industry.

The utilization of twin data enables the linking of devices, as demonstrated in Fig. 3. According to this, servers have the capability to present data regarding equipment, equipment regulations, and regulatory reliability on mobile phones, tablets, and other terminal devices. Furthermore, customers have the ability to comprehend and provide input on the current state of affairs at their convenience. Digital twins primarily encompass tools for modeling, simulation, and data fusion [33]. Additional data sets are incorporated into the data fusion process using modeling and simulation technology. In the realm of industrial manufacturing, the process of altering the design and assembling the many components of a product often requires multiple iterations and consumes significant amounts of both human labor and material resources [34]. Digital twins enable the creation of a virtual environment for industrial manufacturing, allowing engineering designers to closely monitor outward modifications in goods and delve into the intricacies of interior components [35]. Consequently, digital twins have been progressively used in various domains such as manufacturing, industry, cities, battlefields, and other settings, in conjunction with the advancement of simulation technologies [36].

### 2.3 Digital twins in fan manufacturing industry

**Fig. 4:**
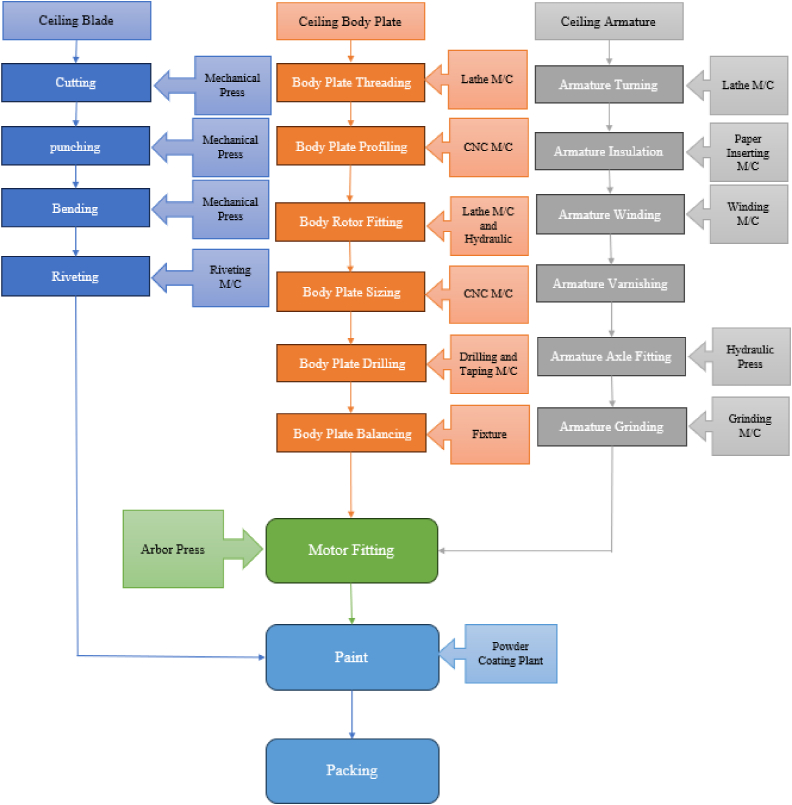
Process flow chart of fan manufacturing industry.

Digital twin technology replicates both the product and manufacturing system in a virtual environment, allowing their digital and physical models to interact and respond dynamically in real time [37]. It offers a robust assurance for the product's smart manufacturing and enhances the speed at which production is integrated with the Internet of Things (IoT). The process flow chart of fan manufacturing industry has been shown in Fig. 4. From the standpoint of product smart manufacturing, the concept of digital twin can be categorized into three levels: unit level (such as equipment-level), system level (such as manufacturing system), and system of system level (such as shop floor-level). This categorization provides a systematic approach to understanding the various uses of digital twins.

Digital twin model used in manufacturing industries can be formulated on five major components including, physical model, virtual model, digital twin data, service, and connections. Digital twin (DT) involves the creation of virtual models that accurately represent physical entities, allowing for the simulation of their activities in a digital environment. The physical world serves as the fundamental basis for digital twins. It encompasses various elements such as devices, products, physical systems, activities, processes, and even organizations. Virtual models should accurately replicate physical entities, encompassing their geometry, attributes, behaviors, and laws. 3D geometric models represent a physical object by specifying its shape, dimensions, tolerance, and structural relationships. The physics model accurately represents the physical phenomena of entities, including deformation, delamination, fracture, and corrosion, based on their physical attributes such as speed, wear, and force. The behavior model explains the actions (such as changes in state, decrease in performance, and coordination) and the corresponding mechanisms that entities use to respond to changes in the external environment. The rule models enable DT to reason, evaluate, and make decisions on its own by following rules that are either derived from domain experts or retrieved from past data.

The use of twin data is a crucial factor in driving the development of digital twins [26]. DT involves handling data that varies over several time scales, dimensions, sources, and is of different types. Data is acquired from physical entities, encompassing both static attribute data and dynamic condition data. Virtual models generate certain data that accurately represents the outcome of a simulation. Data is acquired from services, providing information on the invoke and execution of the service. Data can be categorized as knowledge, which is acquired either through the expertise of topic specialists or by extracting it from pre-existing data. Fusion data refers to the data that is formed through the fusion of all the previously described data.

In the context of the increasing integration of products and services in contemporary society, an increasing number of firms are recognizing the significance of service. To begin, digital twins offer its consumers a variety of application services, including simulation, verification, monitoring, optimization, diagnostic and prognosis, prognostic and health management (PHM), and other related services [38]. Additionally, the construction of a functional digital twin requires the utilization of various external services, including data services, knowledge services, algorithms services, and more. Finally, the functioning of DT relies on the ongoing assistance of diverse platform services that can facilitate tailored software development, model construction, and service provision. Digital models are constantly linked to their corresponding physical counterparts in order to provide sophisticated simulation, operation, and analysis. Interconnections among tangible objects, digital representations, services, and information facilitate the interchange of data and information.

Internal digital twins are primarily concerned with the internal operations of a physical system or object. Data for these systems is gathered by sensors embedded in the actual object itself, which track various component performance metrics such as temperature and pressure. To comprehend how internal components interact and how internal activities might be optimized, employ an internal digital twin. The external digital twin is concerned with the physical object's interactions with the outside world. The data used in the external digital twin is gathered via sensors positioned on the object or in its surroundings. These sensors may record weather information, user input, or interactions with external systems. An external digital twin can be used to optimize an object's interaction with the outside environment and assess how well it performs in real-world scenarios.

### 2.4 enabling technologies for digital twins

**Fig. 5:**
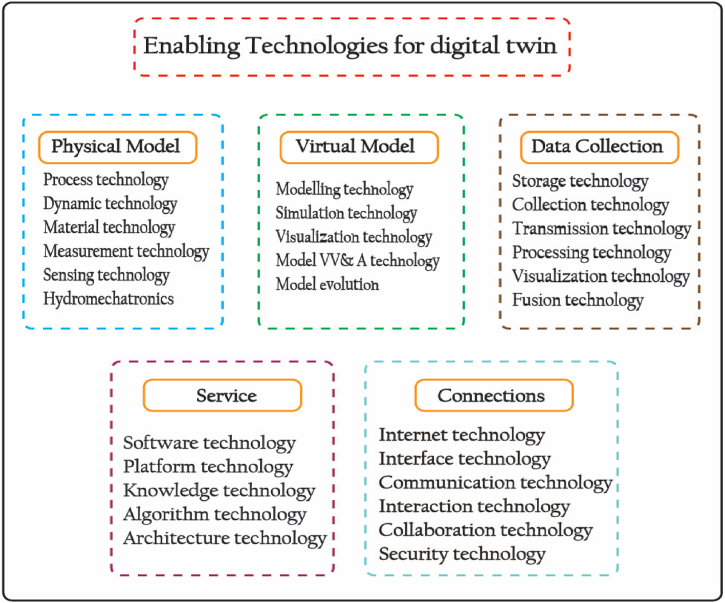
Enabling technologies of digital twin.

The various digital twin modules including physical model, virtual model, data collection, service, and connection require a range of enabling technologies. Fig. 5 depicts the essential enabling technologies for the digital twin. To comprehend the concept of digital twins, a comprehensive comprehension of the physical realm is essential. Digital twin encompasses a wide range of disciplines, including dynamics, structural mechanics, acoustics, thermals, electromagnetic, materials science, hydro mechatronics, and control theory [39]. To improve the accuracy and resemblance of the models, physical items and processes are transferred to virtual space in conjunction with knowledge, sensing, and measurement technologies. A variety of modeling tools are necessary for the virtual model. Technologies for visualization are essential for real-time tracking of processes and physical assets. The precision of virtual models has a direct impact on the efficiency of DT [40]. Thus, it is necessary to validate the model's using verification, validation, and accreditation (VV&A) technologies and optimize them using optimization methods. In addition, the use of simulation and retrospective technologies can facilitate the quick identification of quality flaws and the verification of feasibility. Given the requirement for virtual models to adapt to ongoing changes in the real world, it is necessary to employ model evolution technologies to facilitate the updating of these models.

Digital twin process generates a substantial amount of data. To derive valuable insights from unprocessed data, it is imperative to employ sophisticated data analytics and fusion technologies. The process includes the gathering, transmission, retention, manipulation, integration, and presentation of data. Applications, resources, expertise, and platforms are all part of the digital twin service umbrella. Technology in platform architecture, application software, knowledge, and service-oriented architecture are necessary to provide these services. Last but not least, digital twin's physical model, virtual model, data collection, and service are all linked so that users can communicate and share data. Interaction, cyber-security, interface, communication protocol, and Internet technologies are all part of the link.

### 2.5 Tools used for digital twin technology

**Fig. 6:**
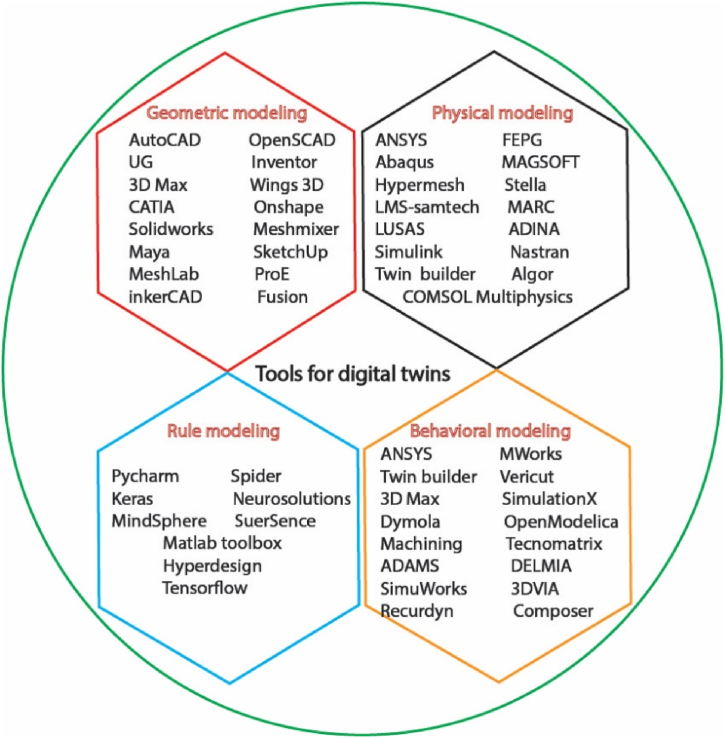
Tools used for digital twins.

Geometric modeling tools are used to precisely define the form, dimensions, positioning, and interconnections of objects. This information is then utilized for tasks such as structural analysis and production planning. SolidWorks is software used for creating three-dimensional models, animating them, generating realistic images, and visualizing them [41]. 3D Max is a software utilized for the creation and refinement of intricate surroundings, and entities (such as individuals, locations, or items), and is extensively employed in commercials, film and television production, manufacturing design, architectural design, 3D modeling, multimedia production, gaming, and several engineering disciplines. The detailed tools used for digital twin technology have been shown in Fig. 6.

To study the physical state of physical goods, physical modeling techniques are used to construct physical models by adding physical properties to geometric models. For instance, sensor data can be utilized by ANSYS's finite element analysis (FEA) software to generate real-time boundary conditions for geometric models and to incorporate wear coefficient or performance deterioration into the models [42]. This can be accomplished by integrating the data into the models. In addition, Simulink may be utilized to develop a model that is based on physics by utilizing multi-domain modeling components [43]. The use of Simulink for physics-based modeling requires the creation of several models, which may include electromechanical, hydraulic, and electrical components. With the use of behavior modeling tools, a model can be created which can adapt to outside forces and disturbances, enhancing digital twin's simulation service performance.

For digital twin modeling, the right software tool is ANSYS Twin Builder, which supports several modeling domains and languages and has a wealth of application-specific libraries [44]. It also can integrate with third-party tools. Twin Builder facilitates the rapid construction, validation, and deployment of digital representations of tangible objects for engineers. Twin Builder's pre-existing libraries offer a wide range of components to construct system dynamics models with the necessary level of detail. These libraries encompass models from many physical domains and fidelity levels. In addition, Twin Builder integrates with ANSYS' physics-based simulation technology to incorporate the intricacies of 3D within the broader systems framework.

## 3 Framework of digital twin-based fan manufacturing industry

**Fig. 7:**
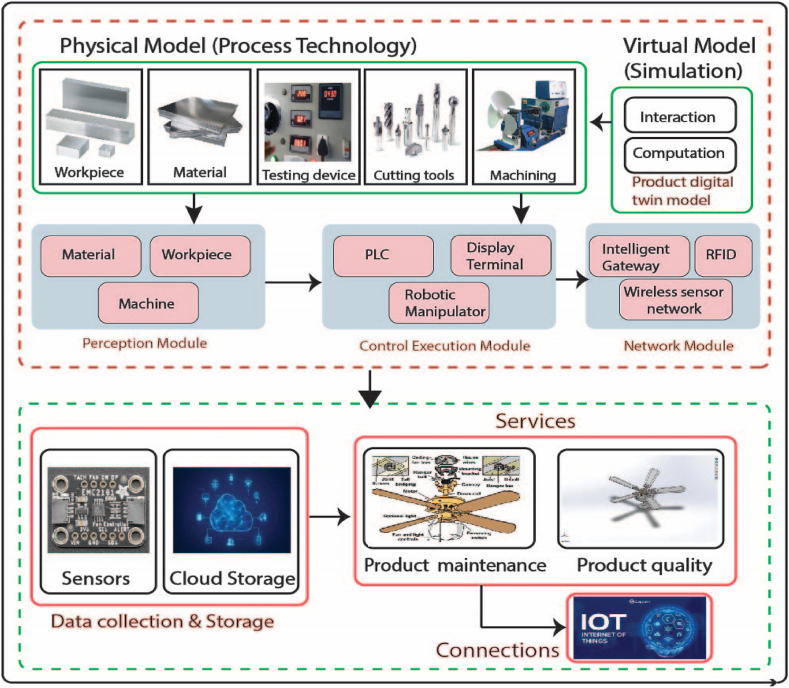
Framework of digital twin-based fan manufacturing industry.

The framework of digital twin-based fan manufacturing industry consists of five components physical model, virtual model, data collection, services, and connections. Fig. 7 illustrates the framework of digital twin-based fan manufacturing industry. The physical layer encompasses the complex, diverse, and ever-changing physical space industrial environment. It alludes to sets of objectively existing physical entities, which mostly consists of machinery used for production and tools for collecting data. Physical machine tools, cutting tools, workpieces, and testing devices are all part of manufacturing machinery. To make completed components, they must supply physical manufacturing data, receive manufacturing tasks, and carry out production operations. Initially, various devices are separated and dispersed, requiring interconnection. Additionally, manufacturing data must be gathered, merged, and enhanced prior to production. Hence, it is imperative to establish a manufacturing Internet of Things (IoT) network in order to achieve the real-time sensing and interconnection of manufacturing resources through network and perception modules. This would enable the iterative optimization of the parts production process by connecting to the virtual model layer.

Specifically, the product digital twin model, the machine digital twin model, the process digital twin model, and so on are all included in the product manufacturing virtual model layer, which is the actual mapping of product manufacturing in cyberspace. The crucial aspect is that the virtual model possesses the properties of interaction, computation, and control. By implementing, virtual model technology several digital twin models become interconnected and cooperative, enabling efficient simulation and analysis of manufacturing operations in cyberspace, such as product machining and quality testing in the physical realm. In addition, the virtual model uses the information layer's knowledge database and machining database to supply optimization methods to the manufacturing service platform, as well as control instructions for the physical model. By continuously predicting, iterating, and optimizing, the digital twin model gains intelligence and becomes more precise throughout the manufacturing process.

Data-driven digital twins have the ability to detect, react to, and adjust to shifting operational and environmental situations. Data can be obtained from various sources such as hardware, software, and networks. Both static attribute data and dynamic status data are part of the hardware data set. Information authentication and real-time perception are two of the most common uses of Internet of Things (IoT) technology such as barcodes, QR codes, RFID, cameras, and sensors [45]. There are two main types of data transmission technologies: wired and wireless. Several methods exist for transmitting data across wires, such as symmetrical and twisted-pair cables, coaxial and fiber optic cables. Wireless transmission can be used for both short and long ranges [46]. Commonly utilized short-range wireless technologies encompass Zig-Bee, Bluetooth, Wi-Fi, Ultra-Wideband (UWB), and Near Field Communication (NFC) [47].

The purpose of storing data is to keep the gathered data for future use in management, analysis, and processing. Database technologies are crucial to data storage. Still, conventional database methods aren't out of the question anymore because of the variety and volume of multisource digital twin data. There has been a recent uptick in interest in big data storage technologies like cloud storage, distributed file systems (DFS), and non-relational database management systems (NoSQL databases) [48].

Services may cover certain resources, including knowledge as well as software and hardware. Technology for generating services encompasses a wide range of applications, from those that perceive and evaluate resources (such as sensors, adapters, and middleware) to those that virtualize and encapsulate resources (such as service-oriented architecture, web services, and semantic services). Some examples of technologies used in service management are search and matching, cooperation, thorough utility evaluation, scheduling, fault tolerance, quality of service (QoS), and so on [49]. In fan manufacturing industry the services can be divided into two parts product maintenance information and product quality information. To accomplish these services various tools such as sensor integration, 3D modeling & Simulation, and digital twin modeling & simulation can be used.

The identification, sensing, and monitoring of products are essential in establishing linkages between physical models, virtual models, data collection and storage, and services. Hence, the utilization of RFID, sensor, wireless sensor network, and other IoT technologies is imperative [50]. Data interchange involves the utilization of communication technology, unified communication interfaces, and protocol technologies. These protocol technologies include protocol parsing and conversion, interface compatibility, and common gateway interface, among others.

## 4 Case study of fan manufacturing industries

The enabling industry 5.0 application is proposed to chief engineers of eight different fan manufacturers out of which five positive responses were gathered and pursued further. Three different avenues were proposed for industry 5.0 implementation as shown in Table 1. The five manufacturers are named FM_1 to FM_5. The three different avenues were production, supply chain and testing transparency. Based on the unanimous decision, the testing transparency is explored.

**Table 1:** Proposed avenues to fan manufacturers.

Avenue	FM_1	FM_2	FM_3	FM_4	FM_5
Production	✗	✗	✗	✓	✗
Supply chain	✗	✗	✓	✗	✓
Testing transparency	✓	✓	✓	✓	✓

### 4.1 Testing transparency

The points raised on testing transparency are.

• Lead times to wait for the results.
• Motor temperature data based on performance data.
• Transparency in experimental testing schedules
• Immediate data sharing with the testing body

To address these concerns the industry 5.0 framework is proposed. With the following features and response of FMs as shown in Table 2. The features starting with “Report” depict the data is going to the FMs. The “obtain” shows that FMs are giving the product model for co-simulation model development. However, the majority did not agree with this concept, a new co-simulation model where fan model is reduced to simple to rotational momentum in the similar volume is explored. There was reluctance in co-simulation model development because of added expertise requirements within the industry and computational costs. The ML performance model has been applauded because of platform independent solution. However, for such ML platform to work, the data from different manufacturers should be used in the developing of the model and is out of scope of current work.

**Table 2:** Features of proposed model and responses of FMs.

Features	FM_1	FM_2	FM_3	FM_4	FM_5
Report RPM of motor	✓	✓	✓	✓	✓
Report Surface temperature of motor	✓	✓	✓	✓	✓
Report order number	✓	✓	✓	✓	✓
Report operational performance	✓	✓	✓	✓	✓
Report test status	✓	✓	✓	✓	✓
Obtain neutral CAD of FAN	✗	✗	✗	✓	✗
Develop Co-Simulation performance model	✗	✓	✓	✓	✓
Develop ML performance model	✓	✓	✓	✓	✓

### 4.2 Proposed framework

**Fig. 8:**
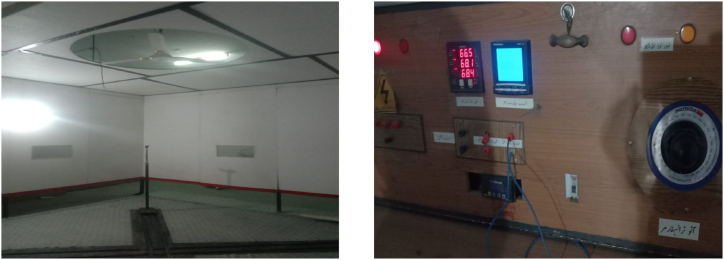
Typical testing setup of fan manufacturing industry.

**Fig. 9:**
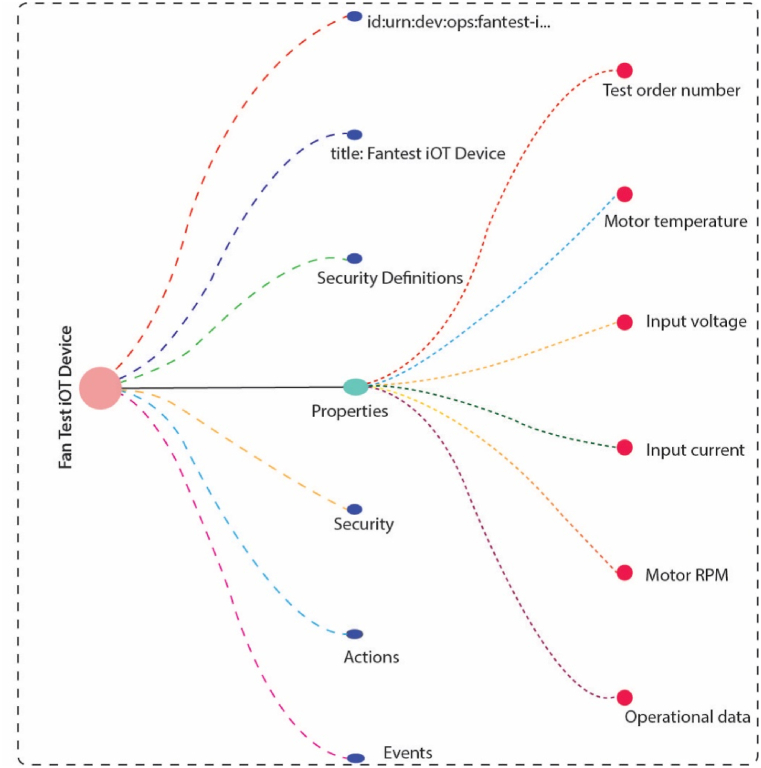
Thing description data model from web of things.

In a fan testing facility, the fan is rotated at various RPMs to gather the operational performance of fan in terms of volumetric air delivery and rated power requirements. The typical testing setup is shown in Fig. 8. The figure shows the fan placement inside the standard room and the measuring instrument is an anemometer that measures the mean air velocity. In this work we added an infrared temperature sensor with the fan hanging point and a tachometer. These devices are linked to an arduino circuit that sends the data at regular intervals to the cloud. The temperature data is crucial to the motor manufacturers and fan manufacturers. Thus, this data is shared with them. The calculated fan power is also shared. This constitutes the physical side of the data collection and sensor linkage. Fig. 9 illustrates the thing description data model from the Web of things which can be used to enable data integration for different FMs.

**Fig. 10:**
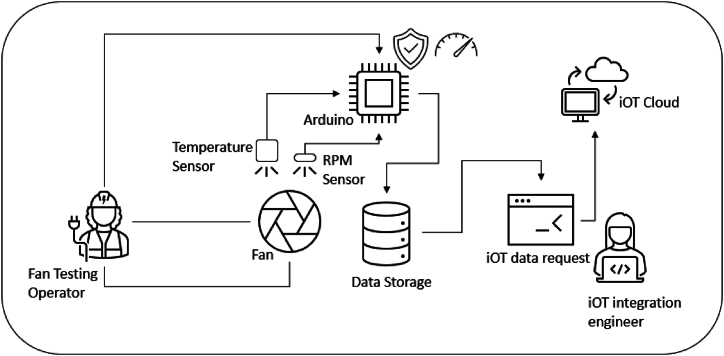
Connection of physical and virtual entities.

Fig. 10 shows the connection of physical and virtual entities. The two-sensor report temperature and RPM, the current and voltage are also reported. For simplicity, the data flow forms the flowrate sensor is omitted. All the data is stored inside the database where FMs can generate custom requests to get their data and perform the calculations.

### 4.3 Test report improvements

**Fig. 11:**
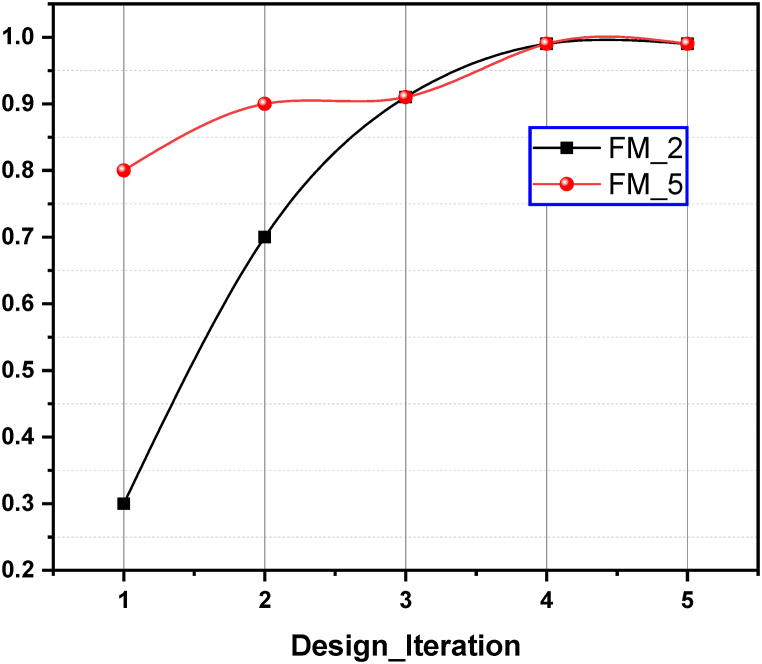
Power factor improvement.

With this implementation, the system sends an email to the company when the test order is set up. Based on the email the company can make online data retrieval thus enabling transparency in the testing system. The temperature, current and voltage data being reported allowed the company to set up their motor test unit to correlate the surface temperature of motor housing with performance. This enabled the improvements in motor design and fan efficiency. The improvements in motor design and dissipation can be presented as the power factor for the design. Upon request from FM_2 and FM_5, the digital twin for motors were generated. For development of this framework, the digital twin of the fan motor is generated in Ansys that predicts the life of the motor based on the motor housing surface temperature. The details of this twin development are out of scope of the current study. As there are different versions of the designs in use by different manufacturers, therefore, the data of motor name was curated and upon setting up the physical test added into the system. The improvements in the design with the help of DT are shown in Fig. 11. The power factor should be close to unity. The improvements in power factor at different design iterations show that 0.99 value of power factor was achieved within five iterations.

**Fig. 12:**
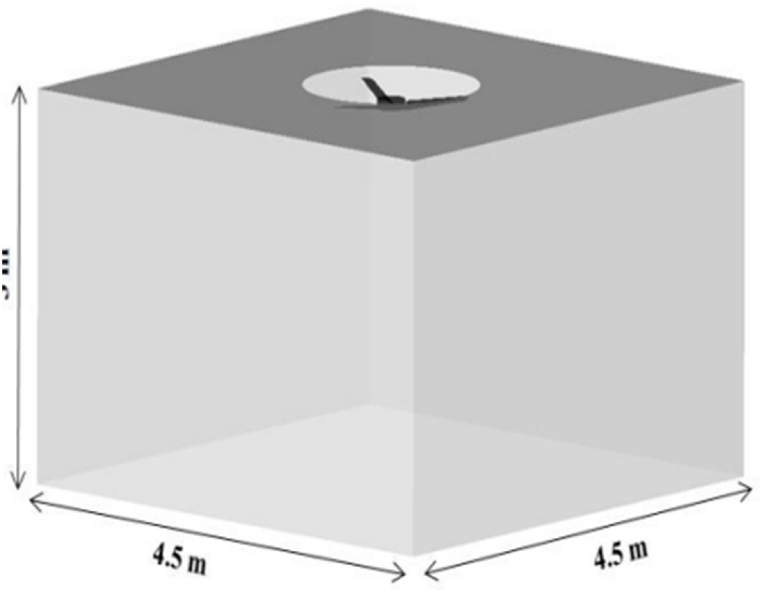
Co-simulation testing setup.

The sharing of neutral file of CAD geometry of the fan to be tested was proposed but it was rejected by the majority of the manufacturers. However, most of the fan blades can be categorized into three angles of the geometry. The co-simulation system is proposed as the testing setup doesn't change and the flow speed at various locations is shared with the manufacturer requiring the test. Thus, developing a simplified fluid model that is fan geometry independent. The typical co-simulation setup is shown in Fig. 12. The different CFD solutions may be utilized depending on the requirements and computational power. The current implementation is based on Ansys CFX. In further, other co-simulation setup may be added.

### 4.4 Operator's response

**Fig. 13:**
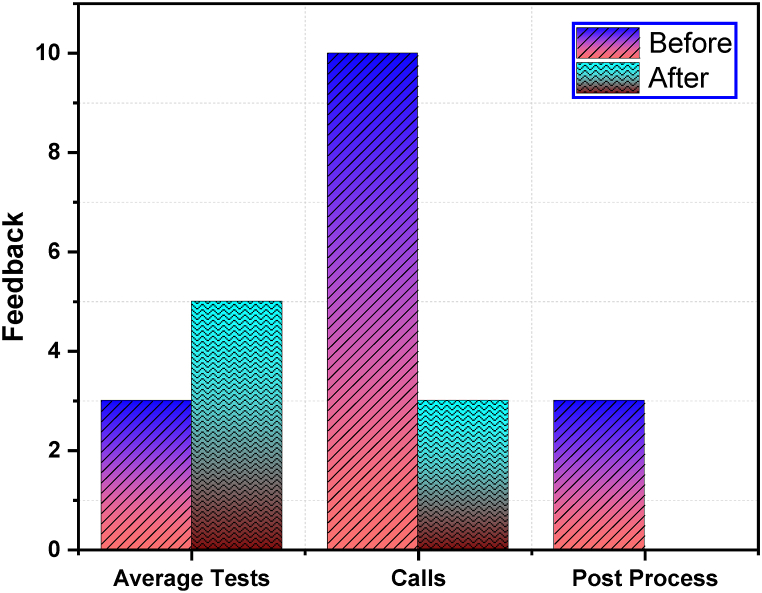
Comparative process improvement.

After the implementation of this system, the operator's feedback was recorded. With the use of phone logs, the difference in phone call frequency per week was calculated before and after the system implementation. The frequency of the phone calls was greatly reduced as shown in Fig. 13. A significant reduction has been observed in phone calls from 10/day to 3/day. The test process was streamlined a lot to reduce the manual scheduling of tests. The operator reported most of the time was lost to reach the safe cooling temperature of the fan housing. After the implementation of the system, the sensor reported when it was safe to change the fan for the next test. The data sharing with the FMs required post processing of the data to an excel file for sharing. This implementation reduced that work post processing work as the FMs were able to check and retrieve the data online. This increases the average number of tests from 3 tests per day to 5 tests per day.

## 5 Conclusions

The exploration of Industry 5.0 in the context of the fan manufacturing industry illuminates a transformative pathway toward sustainable development and enhanced operational efficiency. This study's focus on Digital Twins Technology within Industry 5.0 underscores its potential for revolutionizing manufacturing processes and fostering socio-environmental sustainability. Industry 5.0 presents a paradigm shift from the profit-centric model of Industry 4.0 towards a more human-centric approach, emphasizing the prioritization of human needs, socio-environmental responsibility, and increased resilience. The integration of Digital Twins into manufacturing processes serves as a pivotal enabler for intelligent machining production lines, holding immense promise for the industry's future.

The engagement with chief engineers of various fan manufacturers provided valuable insights into the feasibility and challenges of implementing Industry 5.0 applications. While initial responses were positive, the study encountered industry-wide reluctance regarding data sharing, signaling a barrier to full-scale adoption. Presently, several international standard-setting organizations are directing their focus towards this pivotal area. The National Institute of Standards and Technology (NIST) has initiated an in-depth exploration into this realm with its Draft NISTIR 8356, titled 'Considerations for Digital Twin Technology and Emerging Standards.' This document delves into the intricate technical aspects of digital twin technology, encompassing facets such as data sharing, cybersecurity, and the critical trust issues associated with digital twins. Furthermore, in 2021, the IPC heralded the introduction of IPC-2551, a comprehensive international standard for digital twins. This standard is pivotal in fostering the interoperability of diverse digital data processing modalities that accurately mirror and articulate physical functions. It also explores the requisite data sharing mechanisms essential for the realization of a holistic digital twin framework. Despite encountering obstacles in industry data sharing, our research showcased tangible improvements, notably in process efficiency and safety enhancements. The implementation of Industry 5.0 in a service-oriented environment demonstrated a substantial increase in testing frequency and ensured safer operating conditions for testing operators. These outcomes underscore the potential benefits awaiting industries upon wider acceptance and implementation of Industry 5.0 frameworks.

The absence of strict standards or regulations in the industry 5.0 landscape reveals a dynamic and evolving domain. Despite the existence of tools and approaches for its implementation, the industry's hesitance toward complete adoption remains a significant challenge. The identified improvements, however, emphasize the transformative impact even partial integration can yield, hinting at the enormous potential of embracing Industry 5.0 principles. In conclusion, this study underscores the pivotal role of Industry 5.0, particularly Digital Twins Technology, in revolutionizing fan manufacturing processes. While challenges persist, the demonstrated enhancements in operational efficiency and safety validate the viability and potential benefits of Industry 5.0.

The finds are in accordance with the international needs to having better standards. The literature on industry 5.0 is application oriented rather than that of industry 4.0 where connections are generalized. Therefore, through this study we propose the use of industry 4.0 implementations and set of rules that prove beneficial to human beings while reducing the cost to the industry or keeping it similar. The adoption of industry 5.0 technology is difficult specially when no apparent value is being generated however, data acquired from industry 4.0 may be incorporated in industry 5.0 paradigm for value generation. Here a case study of a fan manufacturing unit is presented, the different types of products are not discussed in this study. The risk of cyberattacks in such environment is not discussed. The future scenario could be what could go wrong in industry 5.0 setup and how it may impact the human centric value chain.

## Data availability statement

The data will be available on demand.

## CRediT authorship contribution statement

**Taoer Yang:** Writing – original draft, Methodology. **Luqman Razzaq:** Software, Investigation, Data curation, Conceptualization. **H. Fayaz:** Visualization, Supervision, Project administration, Funding acquisition. **Atika Qazi:** Writing – review & editing, Validation, Formal analysis.

## Declaration of competing interest

The authors declare that none of the work reported in this study could have been influenced by any known competing financial interests or personal relationships.
